# Nicotine Concentration of E-Cigarettes Used by Youths

**DOI:** 10.1001/jamanetworkopen.2025.2215

**Published:** 2025-03-27

**Authors:** Junhan Cho, Richard A. Miech, Alyssa F. Harlow, Dae-Hee Han, Hongying D. Dai, Steve Sussman, Adam M. Leventhal

**Affiliations:** 1 Department of Population and Public Health Sciences, University of Southern California, Los Angeles; 2 Institute for Addiction Science, University of Southern California, Los Angeles; 3 Institute for Social Research, University of Michigan, Ann Arbor; 4 University of Nebraska College of Public Health, Omaha

## Abstract

This cross-sectional study investigates rates of nicotine concentrations vaped by US youths and associated substance use characteristics.

## Introduction

When US youth vaping peaked in 2019, 5% was the maximum nicotine concentration sold in most e-cigarette products.^1^ Although 5% is already very high, the Food and Drug Administration recently authorized e-cigarettes with ultrahigh concentrations (6%)^2^ and mandates no e-cigarette nicotine concentration limits or labeling requirements. Some packaging labels nicotine concentration, but youths obtain unpackaged devices from third parties without knowing concentrations.^3^ To inform e-cigarette nicotine concentration limits and labeling policies, this study investigated the prevalence of nicotine concentrations that US youths reported vaping; associations of vaping nicotine concentrations at or above 2 thresholds (≥5% vs ≤4% and ≥6% vs 5%) with chronic, frequent vaping and polytobacco and substance use; and association of e-cigarettes obtained from third parties with not knowing nicotine concentration.

## Methods

The Monitoring the Future study^4^ surveyed nationally representative samples of US eighth, tenth, and twelfth grade students February to June 2024 in schools (response rates = 76%-89%). The University of Michigan Institutional Review Board approved this cross-sectional study. Passive (letter to parents or caregivers) or active (written) informed consent was obtained from parents of participants aged younger than 18 years or from participants aged 18 years or older.

Weighted prevalence of self-reported past-30-day usual nicotine concentration vaped (1%-2%, 3%-4%, 5%, ≥6%, and *Don’t know*) were estimated. Prevalences of self-reported e-cigarette and other substance use outcomes were calculated stratified by nicotine concentration. Separate demographics-adjusted log-binomial regressions examined associations of nicotine concentration with each outcome, using predetermined exposure contrasts (≥5% vs ≤4%, ≥6% vs 5%, and does not vs does know nicotine concentration vaped). Analyses used Mplus software version 8.11 (Muthén and Muthén)^5^ with complex survey design options, maximum likelihood estimation accounting for missing demographic covariate data (missingness = 1.8%-7.4%), and Benjamini-Hochberg multiple test correction for maintaining .05 study-wise false discovery. This study is reported following the STROBE reporting guideline. Survey questions and methodological details are listed in the eMethods in Supplement 1.

## Results

Among 2318 youths with past-30-day-vaping nicotine concentration data (18.7% eighth, 32.0% tenth, and 49.4% twelfth grade; 53.6% female; 19.4% Hispanic, 12.0% non-Hispanic Black, 45.0% non-Hispanic White, and 23.6% other non-Hispanic race), 41.4% (95% CI, 38.7%-44.0%) reported not knowing nicotine concentration. Among remaining youths, 20.9% (95% CI, 17.9%-24.4%), 13.5% (95% CI, 11.4%-15.9%), 52.6% (95% CI, 48.1%-57.1%), and 13.0% (95% CI, 10.9%-15.3%) reported vaping 1% to 2%, 3% to 4%, 5%, and ≥6% nicotine, respectively.

All outcomes differed by nicotine concentration (Table 1). In demographic-adjusted contrasts, we found higher odds of vaping more than 20 of 30 days, 5-year vaping expectations, early-age vaping onset, and past-30-day use of most or all tobacco products and polytobacco use in youths vaping 5% or greater vs 4% and 6% or greater vs 5% nicotine concentrations. We found higher odds of vaping-relapse histories and past-30-day alcohol and marijuana use in the first comparison and no difference in non-nicotine substance use in the second comparison. Youths reporting not knowing vs knowing nicotine concentration had higher odds of obtaining e-cigarettes from nonretail third parties (Table 2).

**Table 1.** Nicotine Concentration Prevalence and Characteristics of Study Sample

	Youths, weighted % (95% CI) (N = 2318)^a^					*P* value^b^
	Nicotine concentration, %					
	1-2 (n = 290)	3-4 (n = 183)	5 (n = 700)	≥6 (n = 186)	Does not know (n = 959)	
Nicotine concentration prevalence^c^						
Overall sample (N = 2318)	12.3 (10.7-14.1)	7.9 (6.6-9.4)	30.9 (27.5-34.4)	7.6 (6.4-9.0)	41.4 (38.7-44.0)	NA
In youths who know nicotine concentration usually vaped (n = 1359)	20.9 (17.9-24.4)	13.5 (11.4-15.9)	52.6 (48.1-57.1)	13.0 (10.9-15.3)	NA	NA
E-cigarette and other substance use characteristics^d^						
Vaped nicotine ≥20 of last 30 d	16.8 (12.6-22.0)	34.8 (22.7-49.2)	60.4 (56.0-64.5)	71.0 (62.8-78.0)	27.0 (23.1-31.4)	<.001
History of failed quit vaping attempts^e^	15.1 (9.9-22.4)	33.8 (24.1-45.1)	40.9 (33.2-49.1)	46.9 (37.7-56.4)	23.6 (19.5-28.4)	<.001
Expects to be vaping in 5 y^e^	3.3 (1.5-6.9)	5.7 (2.3-13.6)	10.8 (7.6-15.1)	20.8 (13.6-30.5)	6.2 (4.5-8.7)	<.001
Obtained e-cigarettes from third party^f^	81.7 (75.5-86.7)	72.5 (59.5-82.6)	58.1 (52.3-63.7)	68.8 (61.1-75.5)	82.9 (79.1-86.1)	<.001
Early age vaping onset (≤grade 7)^g^	40.8 (30.0-52.5)	52.9 (36.3-68.9)	56.6 (48.4-64.5)	68.4 (55.2-79.2)	43.0 (35.6-50.8)	.001
Past-30-d use						
Cigarettes	4.3 (2.1-8.7)	8.3 (4.5-14.8)	14.5 (11.1-18.6)	24.3 (17.6-32.6)	8.2 (5.6-11.8)	<.001
Cigars or cigarillos^g^	2.9 (1.0-8.1)	4.0 (1.0-14.8)	14.8 (11.0-19.7)	31.4 (19.1-47.0)	4.8 (2.6-8.6)	<.001
Nicotine gummies, mints, or pouches^g^	5.1 (2.3-10.9)	17.5 (7.8-34.4)	19.0 (13.6-25.7)	34.4 (22.5-48.6)	13.2 (8.5-19.9)	.003
Smokeless tobacco^g^	5.9 (2.4-13.9)	NA^h^	9.5 (6.1-14.5)	26.1 (14.4-42.7)	5.3 (2.9-9.4)	<.001
Non–e-cigarette tobacco products, total No.^g^^,^^i^						
≥1 vs No use	14.4 (7.4-26.1)	28.6 (17.8-42.6)	37.8 (29.5-46.8)	53.5 (39.5-66.6)	25.2 (17.2-35.4)	.001
≥2 vs No use	6.1 (2.9-12.5)	11.3 (3.8-28.9)	19.5 (14.4-25.8)	34.8 (22.4-49.8)	8.1 (5.1-12.7)	<.001
≥3 vs No use	NA^h^	NA^h^	9.7 (5.8-15.8)	26.8 (15.7-41.9)	4.4 (2.3-8.1)	<.001
Alcohol	39.3 (32.5-46.6)	56.0 (46.1-65.4)	62.1 (56.5-67.5)	63.2 (53.3-72.1)	46.1 (41.8-50.4)	<.001
Marijuana	45.0 (38.4-51.8)	56.1 (47.1-64.7)	62.1 (57.7-66.4)	69.3 (56.6-79.5)	52.7 (48.0-57.4)	<.001

Abbreviation: NA, not applicable.

^a^ Outcomes are given by categories of nicotine concentration typically used in an e-cigarette vaping device in the past 30 days. Questionnaire measures appear in the eMethods in Supplement 1.

^b^ From χ^2^ test of omnibus group differences. All outcomes in this table were considered statistically significant at a 2-sided *P* = .05 after Benjamini-Hochberg correction for multiple tests to maintain studywise false discovery rate.

^c^ Row percentages.

^d^ Column percentages with positive status for respective binary outcome; population numbers range from 2231 to 2318 unless otherwise specified.

^e^ Population numbers range from 1458 to 1463.

^f^ Most recent source of obtaining e-cigarettes was from a relative, friend, illegal e-cigarette “dealer,” or other noncommercial source.

^g^ Population numbers range from 708 to 914.

^h^ Estimate not presented due to cell size <5.

^i^ Total number of following used: large cigars, little cigars or cigarillos, hookah, smokeless tobacco, nicotine gummies or mints, nicotine pouches, and cigarettes.

**Table 2.** Associations of E-Cigarette Nicotine Concentration With Other Characteristics

Characteristic	≥5% vs ≤4% Nicotine^a^		≥6% vs 5% Nicotine^a^		Does not know vs knows nicotine concentration^a^	
	aRR (95% CI)	*P* value^b^	aRR (95% CI)	*P* value^b^	aRR (95% CI)	*P* value^b^
E-cigarette use characteristics^c^						
Vaped nicotine ≥20 of the last 30 d	4.46 (3.15-6.33)	<.001	2.56 (1.54-4.22)	<.001	0.41 (0.30-0.56)	<.001
History of failed quit vaping attempts	2.71 (1.74-4.21)	<.001	1.52 (0.91-2.54)	.11	0.57 (0.44-0.74)	<.001
Expects to be vaping in 5 y	3.12 (1.45-6.73)	.004	2.34 (1.27-4.30)	.006	0.71 (0.48-1.05)	.09
Obtained e-cigarettes from third party	0.55 (0.35-0.87)	.01	1.30 (1.03-1.63)	.03	2.30 (1.68-3.15)	<.001
Early-age vaping onset (≤grade 7)	4.08 (2.22-7.50)	<.001	1.21 (0.65-2.24)	.54	0.52 (0.40-0.68)	<.001
Past-30-d use of non–e-cigarette substances^e^						
Combustible cigarettes	2.91 (1.85-4.56)	<.001	1.86 (1.21-2.85)	.01	0.70 (0.54-0.90)	.01
Cigars or cigarillos	6.58 (2.59-16.68)	<.001	3.09 (1.38-6.88)	.006	0.36 (0.17-0.80)	.01
Nicotine gummies, mints, or pouches	2.71 (1.62-4.55)	.001	1.96 (1.22-3.16)	.01	0.81 (0.49-1.35)	.41
Smokeless tobacco products, No.	2.22 (0.94-5.21)	.07	2.59 (1.01-6.70)	.04	0.55 (0.19-1.55)	.25
≥1 vs 0	2.54 (1.55-4.14)	<.001	1.82 (1.16-2.86)	.01	0.72 (0.50-1.02)	.07
≥2 vs 0	4.41 (2.31-8.39)	<.001	2.25 (1.01-4.97)	.04	0.44 (0.20-0.97)	.04
≥3 vs 0	5.25 (2.04-13.46)	.001	3.75 (2.08-6.76)	<.001	0.41 (0.11-1.51)	.18
Alcohol	1.51 (1.12-2.03)	.007	1.13 (0.81-1.56)	.47	0.68 (0.56-0.82)	<.001
Marijuana	2.01 (1.56-2.58)	<.001	1.47 (0.86-2.52)	.16	0.78 (0.64-0.96)	.02

Abbreviation: aRR, adjusted risk ratio.

^a^ The aRR with 95% CI is given for the association of nicotine concentration (percentage nicotine reported typically vaping in the past 30 days) pairwise contrast with the respective binary outcome from log-binomial regression models including school grade, sex, self-reported race and ethnicity from surveys, population density, 4-year college plan, and parental education covariates. Demographic variables are detailed in the eMethods in Supplement 1. Participants self-reported race and ethnicity on the survey for descriptive purposes based on fixed categories: Asian, Black, Hispanic, White, multiracial, and another race or ethnicity (combined due to small sizes: American Indian and Alaska Native, Native Hawaiian or Other Pacific Islander, and Middle Eastern).

^b^ Outcomes were considered statistically significant at a 2-sided *P* = .05 after Benjamini-Hochberg correction for multiple tests to maintain studywise false discovery rate.

^c^ Outcome variable definitions and sample sizes appear in a Table 1 note, and questionnaire items appear in the eMethods in Supplement 1.

## Discussion

This cross-sectional study found that in 2024, most US youths with past-30-day vaping reported using e-cigarettes with very high nicotine concentrations or not knowing the concentration. We observed dose response–like associations of nicotine concentration with frequent, chronic vaping and polytobacco use. Youths who vaped ultrahigh (≥6%) vs very high (5%) nicotine e-cigarettes had incrementally increased risk of using various nicotine products and frequent, chronic vaping patterns but did not differ in other substance use. Hence, associations with vaping ultrahigh nicotine concentrations were nicotine-specific and not explained by generalized propensity to use all substances. Youths reporting not knowing their nicotine concentration more commonly obtained e-cigarettes from third parties and had lower-risk vaping and tobacco-use profiles. Those associations may reflect that inexperienced vapers paid less attention to nicotine concentration or were less inclined to obtain their own prepackaged, labeled devices from retailers.

Study limitations include unknown impacts of self-report error and misclassification, absenteeism, inattention to e-liquid volume and other parameters that differentiate nicotine exposure, and cross-sectional design. Future research can use longitudinal designs to determine directionality of associations and leverage nicotine constituent analysis of e-cigarette devices to classify concentration.

In 3 US states and the European Union, selling e-cigarettes with nicotine concentrations exceeding prespecified limits is prohibited. Federal policies require alcoholic beverage labels to list alcohol concentration. Requiring on-device nicotine concentration labels and warnings and limiting high-nicotine e-cigarette sales could protect US youths.

## Notes

Supplement 1.
eMethods.

Supplement 2.
Data Sharing Statement

## References

[1] Duell AK, Pankow JF, Peyton DH. Nicotine in tobacco product aerosols: ‘it’s déjà vu all over again’. Tob Control. 2020;29(6):656-662. doi:10.1136/tobaccocontrol-2019-055275 31848312 PMC7591799

[2] US Food and Drug Administration. Premarket Tobacco Product Marketing Granted Orders. Updated March 28, 2024. Accessed November 19, 2024. https://www.fda.gov/tobacco-products/premarket-tobacco-product-applications/premarket-tobacco-product-marketing-granted-orders

[3] Alexander JP, Williams P, Lee YO. Youth who use e-cigarettes regularly: a qualitative study of behavior, attitudes, and familial norms. Prev Med Rep. 2018;13:93-97. doi:10.1016/j.pmedr.2018.11.011 30568866 PMC6290379

[4] Miech RA, Johnston LD, Patrick ME, O’Malley PM. Monitoring the Future: National Survey Results on Drug Use, 1975-2023: Overview and Detailed Results for Secondary School Students. University of Michigan Institute for Social Research; 2024. Accessed February 13, 2025. https://monitoringthefuture.org/wp-content/uploads/2024/01/mtfoverview2024.pdf

[5] Muthén LK, Muthén BO. Mplus: Statistical Analysis with Latent Variables: User’s Guide. Version 8. Muthén & Muthén; 2017. Accessed February 13, 2025. https://www.statmodel.com/HTML_UG/introV8

