# Supplementary material for: Nicotine Concentration of E-Cigarettes Used by Youths
Source: JAMA Netw Open. 2025 Mar 27;8(3):e252215. doi: 10.1001/jamanetworkopen.2025.2215 (PMC11950894; doi:10.1001/jamanetworkopen.2025.2215)
Supplement: Supplement 1. — eMethods. [file jamanetwopen-e252215-s001.pdf]

Supplemental Online Content

Cho J, Miech RA, Harlow AF, et al. Nicotine concentration of e-cigarettes used by youths. *JAMA Netw Open*. 2025;8(3):e252215. doi:10.1001/jamanetworkopen.2025.2215

eMethods.

This supplemental material has been provided by the authors to give readers additional information about their work.

© 2025 Cho J et al. *JAMA Network Open*.

eMethods

About the Monitoring the Future (MTF) study

The MTF study conducts repeated cross-sectional annual surveys of nationally-representative samples of 8th, 10th, and 12th grade students every year since 1975 to provide surveillance on the prevalence of use of addictive drugs and other outcomes in US youth. Each year's data collection takes place in approximately public and private high schools and middle schools selected to provide an accurate representative cross section of students throughout the coterminous United States at each grade level. A multi-stage random sampling procedure is used for securing the nationwide sample of students each year at each grade level. Stage 1: The selection of particular geographic areas. Stage 2: The selection (with probability proportionate to size) of one or more schools in each area. Stage 3: The selection of classes within each school. Within each school, up to 350 students may be included. In schools with fewer students, the usual procedure is to include all of them in the data collection. In larger schools, a subset of students is selected either by randomly sampling entire classrooms or by some other random method that is judged to be unbiased. Sampling weights are used when the data are analyzed to correct for unequal probabilities of selection that occurred at any stage of sampling.

About 10 days before the administration, the students are given flyers explaining the study. Also, advance letters to parents inform them about the study and provide them means for declining their child's participation if they so desire. The actual questionnaire administrations are conducted by the study staff, following standardized procedures detailed in a project instruction manual. The questionnaires are group administered in classrooms during a normal class period whenever possible; however, circumstances in some schools require the use of larger group administrations. Students absent from school on survey days are not included.

MTF 2024 study methodological details germane to this study

In 2024, 272 schools participated in the MTF study in total (97 8th grade schools, 93 10th grade schools, 82 12th grade schools). While most questions are administered to the entire sample of MTF respondents, some questions are administered to randomly selected subset in most years, which was the case in 2024. In 2024, the history of unsuccessful quit vaping attempts and anticipating vaping outcomes measures were administered to all 8th and 10th graders and to a randomly selected two-thirds of 12th graders. For the cigars/cigarillos, smokeless tobacco, nicotine gummies/mints, and poly-tobacco use measures, these were administered to a randomly selected one-fourth of 8th and 10th graders and randomly selected one-third of 12th graders. The remaining measures in this study were administered to all students. The demographic covariates and their categories in this study included grade (8th[reference], 10th, 12th), population density of school location (city[reference], suburban/town, rural), and self-reported sex (male[reference], female, other/prefer not to answer), race/ethnicity (NH-White[reference], Hispanic/Latino, NH-Black, Another race/ethnicity), plans to go to a 4-year college (yes/no), and having ≥1 parents who attended college (yes/no).

Questionnaire items in this study

1. In the LAST 30 DAYS, what amount of nicotine did you usually use in your electronic vaping device?
   - *Response options:* 1="1 - 2%" 2="3 - 4%" 3="5%" 4="6% or higher" 8="Don't know"
   - *Coding*: Raw responses, and see Table 2 note

2. On how many DAYS (if any) during the LAST 30 DAYS have you vaped nicotine?
   - *Response options:* 1="0 Days" 2="1-2 Days" 3="3-5 Days" 4="6-9 Days" 5="10-19 Days" 6="20-29 Days" 7="30 Days"
   - *Coding*: Responses 6 and 7 = positive for outcome

3. Have you ever tried to stop vaping nicotine and found that you could not?
   - *Response options:* 1="Yes" 2="No"
   - *Coding*: Raw responses

4. Do you think you will be vaping nicotine FIVE YEARS from now?
   - *Response options:* 1="I definitely will" 2="I probably will" 3="I probably will not" 4="I definitely will not"
   - *Coding:* Response 1 = positive for outcome

5. The last time you used a vaping device how did you get it?
   - *Response options:* 0="Took from a relative without asking" 1="Given for free by a friend" 2="Given for free by a relative " 3="Bought from a friend" 4="Bought from a relative " 5="At a convenience store (such as 7-Eleven) or a gas station" 6="At a "vape" store" 7="Over the internet " 8="From a person who is known to sell vaping devices to kids (a dealer)" 9="Other [V7884.TEXT capture write in]"
   - *Coding:* Responses 0, 1, 2, 3, 4, 8, and 9 = positive for outcome

6. When (if ever) did you FIRST vape an e-liquid with nicotine.
   - *Response options*: 1="Grade 4 or below" 2="Grade 5" 3="Grade 6" 4="Grade 7" 5="Grade 8" [10th grade only: 6="Grade 9" 7="Grade 10"] for 8th and 10th graders; 1="Grade 6 or below" 2="Grade 7" 3="Grade 8" 4="Grade 9" 5="Grade 10" 6="Grade 11" 7="Grade 12" for 12th graders
   - *Coding:* Responses 1, 2, 3, and 4 for 8th/10th graders and 1 and 2 for 12th graders for 12th graders = positive for outcome

© 2025 Cho J et al. *JAMA Network Open*.

7**.** During the LAST 30 DAYS, on how many DAYS (if any) have used . . .

a. …large cigars?
b. …flavored little cigars or cigarillos?
c. …regular little cigars or cigarillos?
d. …tobacco using a hookah (water pipe)?
e. …smokeless tobacco (chewing tobacco, snuff, plug, dipping tobacco, snus)?
f. …nicotine gummies?
g. ...nicotine hard candy or nicotine mints?

- *Response options*: 1="None" 2="1-2 days" 3="3-5 days" 4="6-9 days" 5="10-19 days" 6="20-30 days"
- *Coding*: Responses 2, 3, 4, 5, and 6 = positive for outcome

8. On how many occasions have you used nicotine pouches during the last 30 days?

- *Response options:* 1="0 Occasions" 2="1-2 Occasions" 3="3-5 Occasions" 4="6-9 Occasions" 5="10-19 Occasions" 6="20-39 Occasions" 7="40 or More"
- *Coding*: Responses 2, 3, 4, 5, 6, and 7 = positive for outcome

Nicotine gummies, mints, and pouches composite variable:

- Coding: positive for outcome if positive for 6f and/or 6g and/or 7

Cigar/cigarillos composite variable:

- Coding: positive for outcome if positive for 6a, 6b, and/or 6c

Total number of non-e-cigarette tobacco products composite variable:

- Coding: total number of positive for outcomes 6a-6g, and 7, recoded as 1+ (v 0); 2+ (v 0); and 3+ (v 0)

9 How frequently have you smoked cigarettes during the PAST 30 DAYS?

- *Response options*: 1="Not at all" 2="Less than one cigarette per day" 3="One to five cigarettes per day" 4="About one-half pack per day" 5="About one pack per day" 6="About one and one-half packs per day" 7="Two packs or more per day"
- *Coding:* Responses 2, 3, 4, 5, 6, and 7 = positive for outcome

10. On how many DAYS during the LAST 30 DAYS have you used alcohol?

- *Response options*: 1="0 Days" 2="1-2 Days" 3="3-5 Days" 4="6-9 Days" 5="10-19 Days" 6="20-29 Days" 7="30 Days"
- *Coding:* Responses 2, 3, 4, 5, 6, and 7 = positive for outcome

11. The next questions are about marijuana or cannabis (sometimes called pot, weed, or hashish). On how many occasions (if any) have you used marijuana (smoking, vaping, edibles) during the last 30 days?

- *Response options*: 1="0 Occasions" 2="1-2 Occasions" 3="3-5 Occasions" 4="6-9 Occasions" 5="10-19 Occasions" 6="20-39 Occasions" 7="40 or More"
- *Coding:* Responses 2, 3, 4, 5, 6, and 7 = positive for outcome

© 2025 Cho J et al. *JAMA Network Open*.
