# Supplementary material for: Nicotine Concentration of E-Cigarettes Used by Youths
Source: JAMA Netw Open. 2025 Mar 27;8(3):e252215. doi: 10.1001/jamanetworkopen.2025.2215 (PMC11950894; doi:10.1001/jamanetworkopen.2025.2215)
Supplement: Supplement 2. — Data Sharing Statement [file jamanetwopen-e252215-s002.pdf]

Data Sharing Statement

Cho. Nicotine Concentration of E-Cigarettes Used by Youths. *JAMA Netw Open*. Published online March 27, 2025. doi:10.1001/jamanetworkopen.2025.2215

Data

Data available: Not right away. The US Monitoring the Future makes data publicly available; however, there is usually a 18 month lag between data collection and making the data publicly downloadable. Because the data are currently restricted until then, there will be a delay. If there is a specific request for the data is made before then, requests to the second author who is the Director the Monitoring the Future Study should be made.
